# Tryptophan metabolism: Mechanism-oriented therapy for neurological and psychiatric disorders

**DOI:** 10.3389/fimmu.2022.985378

**Published:** 2022-09-08

**Authors:** Dan Li, Shuang Yu, Yu Long, Ai Shi, Jie Deng, Yin Ma, Jing Wen, Xiaoqiu Li, Songyu Liu, Yulu Zhang, Jinyan Wan, Nan Li, Rui Ao

**Affiliations:** ^1^ State Key Laboratory of Southwestern Chinese Medicine Resources, Chengdu University of Traditional Chinese Medicine, Chengdu, China; ^2^ Oncology Center, Sichuan Provincial People's Hospital, Chengdu, China

**Keywords:** tryptophan metabolism, neurotoxicity, neuroprotection, influence factor, neurological, psychiatric disorders

## Abstract

Neurological and psychiatric disorders are a category of chronic diseases that are widespread and pose serious mental and physical health problems for patients. The substrates, products, and enzymes of Tryptophan metabolism all contribute to the development of neurological and psychiatric disorders. This paper deals with three metabolic pathways of tryptophan that produce a series of metabolites called tryptophan Catabolics (TRYCATs). These metabolites are involved in pathological processes such as excitotoxicity, neuroinflammation, oxidative stress, and mitochondrial damage and are closely associated with neurological and psychiatric disorders such as Alzheimer’s disease and depression. Here, we review the elements that affect how tryptophan metabolism is regulated, including inflammation and stress, exercise, vitamins, minerals, diet and gut microbes, glucocorticoids, and aging, as well as the downstream regulatory effects of tryptophan metabolism, including the regulation of glutamate (Glu), immunity, G-protein coupled receptor 35 (Gpr35), nicotinic acetylcholine receptor (nAChR), aryl hydrocarbon receptor (AhR), and dopamine (DA). In order to advance the general understanding of tryptophan metabolism in neurological and psychiatric disorders, this paper also summarizes the current situation and effective drugs of tryptophan metabolism in the treatment of neurological and psychiatric disorders and considers its future research prospects.

## Graphical Abstract

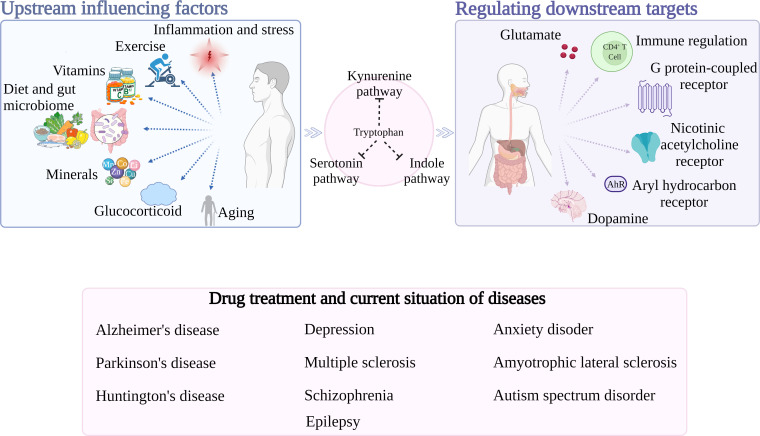

## 1 Introduction

According to the World Health Organization (WHO), approximately 6.8 million people worldwide die each year from a variety of neurological and psychiatric disorders, including Stroke, Alzheimer’s disease (AD), and Parkinson’s disease (PD). Not only are neurological and psychiatric disorders expensive to treat, but patients also experience significant stigma, social exclusion, and poor quality of life (1). Neurological and psychiatric disorders can be caused by external stresses and internal genetic factors. Initial mood disorders, if left untreated, can gradually cause changes in brain physiological activity and eventually develop into neurological and psychiatric disorders (2). The prevalence of mental disorders is increasing worldwide year by year, and the disease burden of mental disorders ranks first among chronic diseases and is one of the three leading causes of disability (3). In the face of the severe personal and social burden caused by neurological and psychiatric disorders, efforts should be made to provide solutions to benefit patients. In order to fulfill the demand for disease-oriented therapies, mechanism-oriented therapies must be implemented on the supply side (4). Mechanism-oriented therapy is the basis and the means, while disease-oriented therapy is the end and the way out, and they are not contradictory. At the same time, focusing on mechanism-oriented therapy can address the co-morbidity of diseases and ultimately achieve the goal of meeting clinical needs.

Currently, the tryptophan metabolic pathway is considered to be a major pathway connecting multiple systems such as the immune inflammatory response of the nervous system, involving stress, inflammation, the kynurenine pathway, 5-HTergic, and glutamatergic neurotransmission (5). Various enzymes or products of the tryptophan metabolic process are extremely closely related to neurological disorders (Alzheimer’s disease, Parkinson’s disease, Huntington’s disease, Amyotrophic lateral sclerosis, Multiple sclerosis, Autism, Epilepsy) and psychiatric disorders (Depression, Schizophrenia, Bipolar disorder, Anxiety) with a high degree of co-morbidity (6). The study of its effect on upstream and downstream is conducive to a holistic understanding of the mechanism of action of this pathway, and provides support for the realization of drug development from mechanism-oriented therapy to disease-oriented therapy. To this end, we outline several metabolic pathways and metabolites of tryptophan, as well as the upstream and downstream influences on tryptophan metabolism. Finally, effective drugs and research advances in tryptophan metabolism in neuropsychiatric disorders are summarized.

## 2 Tryptophan

Tryptophan (TRP), an alfa-amino acid with the chemical name 2-amino-3-(1H-indol-3-yl) propanoic acid, may exist as L, D, or DL isomers (7). It is an essential amino acid for protein synthesis, which is the least abundant in cells and proteins (8). It can only be obtained through diet and is the substrate of several bioactive compounds with important physiological effects (9). TRP is metabolized to various bioactive compounds through different metabolic pathways, which in turn participate in various physiological activities in the body and regulate body homeostasis. These TRYCATs include kynurenine(KYN), serotonin, melatonin, indole, tryptamine, vitamin B3, protein, etc (10). However, only free TRP can participate in metabolism and unbound TRP can be transported across the blood-brain barrier (BBB) *via* non-specific and competitive Large neutral amino acid transporter (LAT). As the only amino acid that can bind to albumin, TRP is first bound to plasma albumin and then released from albumin in the presence of free fatty acids to function (11). In general, 85-90% of TRP is present in the plasma in the bound form. By competitively binding to albumin, substances like free fatty acids can replace TRP and raise the concentration of free TRP in the body.

## 3 Tryptophan metabolic pathway

**Figure 1:**
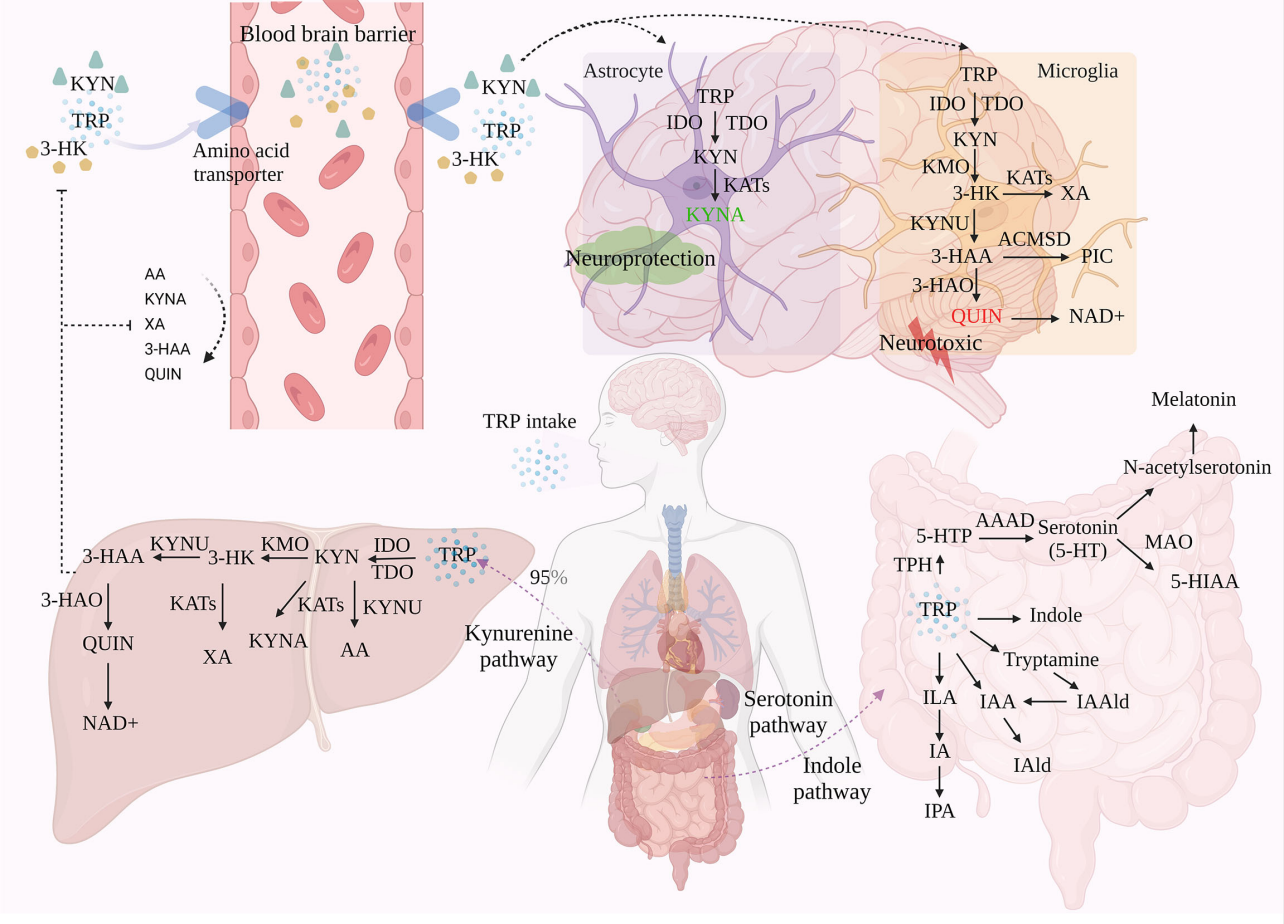
The three metabolic pathways of TRP. TRP is metabolized through three different pathways: KP, SP, and IP, with KP accounting for around 95% of TRP metabolism in the liver. Under the action of a series of enzymes, TRP generates metabolites such as KYN and KYNA *via* KP. Of these, only TRP, KYN, and 3-HK can enter the brain through the BBB, which in turn is involved in the neurotoxic and neuroprotective branches of TRP. A small proportion of TRP is metabolized in the gastrointestinal tract *via* SP and IP.

TRP is metabolized through three pathways, the kynurenine pathway (KP), the serotonin pathway (SP), and the indole pathway (IP). Imbalances in TRP metabolic pathways, particularly an excess or change in the ratio of metabolites of specific neuroactive features, are thought to be responsible for a variety of neurological and psychiatric disorders (**Figure 1**).

### 3.1 Kynurenine pathway

In the metabolism of TRP, 95-99% of TRP is metabolized toward the KP. Through signaling cascade reaction, TRP is catalyzed by indoleamine 2, 3-dioxygenase (IDO) and tryptophan 2, 3-dioxygenase (TDO), the first rate-limiting enzymes of the tryptophan metabolic pathway, to produce N-formyl-kynurenine, followed by the formation of KYN in the presence of formamidase (12, 13). Not only is KYN the first stable product of KP, but it is also the central point of KP. 60% of KYN in the central nervous system (CNS) is derived from peripheral KYN carried by LAT across the BBB and degraded in the CNS *via* two cellular pathways, astrocytes and microglia (14). This pathway is involved in inflammation, immune response, and excitatory neurotransmission, and is associated with a variety of neurological and psychiatric disorders, as well as with liver and kidney dysfunction, cataracts, diabetes, and various chronic malignant diseases and abnormal pregnancies (15). Modern pharmacological studies have revealed that metabolites of KYN can modulate glutamatergic neurotransmission. In particular, they exert neurotoxic/neuroprotective effects through the antagonist/agonist activity of the N-methyl-D-aspartate receptor (NMDAR). Therefore, KP is considered to be a bridge between systemic inflammation and tissue and organ function.

TDO is found in eukaryotes and bacteria, but IDO is only found in mammals and yeast, although mammals can express both IDO and TDO (16). IDO (IDO-1, IDO-2) is considered to be the main catalytic enzyme for KYN production under inflammation or stress, and can be activated by various pro-inflammatory factors such as interferon (IFN-γ), lipopolysaccharide (LPS), and tumor necrosis factor (TNF), which in turn accelerate KP (17). Among them, IDO-1 is widely expressed in immune and non-immune tissues other than the liver, including in immune cells such as microglia, astrocytes, and macrophages (11). Activation of IDO-1 may be associated with activation of NF-κB and MAPK signaling pathways by inflammatory factors and release of IFN-γ by activated CD4^+^ T cells (18). IDO-2 is mainly expressed in the liver, kidney, and epididymis of mice and catalyzes the same substrates as IDO-1, but its activity is much lower than IDO-1. TDO, on the other hand, is present in large amounts in the liver and is preferentially activated by glucocorticoids, but also by low concentrations of reactive oxygen species (ROS), however, accumulation of ROS inactivates TDO. Overall, TDO is only responsible for the metabolism of KP in normal physiological situations, while IDO acts more in pathological situations.

It is generally accepted that TRP is metabolized to quinolinic acid (QUIN) in microglia and macrophages, or kynurenic acid (KYNA) in astrocytes. Depending on the activity of the metabolite, this reaction is considered to be “neuroprotective” in astrocytes and “neurotoxic” in microglia and macrophages, thus achieving a duality in terms of disease. Therefore, QUIN levels and the QUIN/TRP ratio are often used as important indicators of pathological changes in neurocentral diseases. Among them, QUIN is an important metabolite of the KP pathway that severely contributes to neuronal cell death and chronic dysfunction through at least nine different mechanisms, including ROS production, disruption of the BBB, glutamate (Glu) excitotoxicity, cytoskeletal instability, mitochondrial dysfunction, promotion of tau protein phosphorylation, and disruption of autophagy (19, 20).In conclusion, KYN has three different metabolic pathways due to different enzymes, conversion to 3-hydroxykynurenine (3-HK) *via* kynurenine 3-monooxygenase (KMO), dehydrogenation to KYNA *via* kynurenine aminotransferase (KAT), and degradation of kynureninase (KYNU) to anthranilic acid (AA), the ratio of different kynurenine metabolite concentrations has been widely used as an indicator of the enzymatic activity of different pathways (21). Currently, TRP, KYN, and 3-HK have been shown to enter the brain *via* the BBB and subsequently produce kynurenine metabolites in activated macrophages and microglia (21). The rate of their transport through the BBB depends on the concentration of LAT in the blood.

#### 3.1.1 Neurotoxic branch

KMO (located in the outer mitochondrial membrane), the main synthetase of the QUIN pathway, converts kynurenine to 3-HK. This component crosses the BBB and increases the substrate required for QUIN production, producing neuronal apoptosis, free radical generation, and oxidative stress damage in the brain at nanomolar concentrations, leading to elevated metal toxicity in rat astrocyte cultures and effectively synergizing the neurotoxic effects of QUIN (22). Subsequently, 3-HK is formed into 3-hydroxyanthranilic acid (3-HAA) by the action of KYNU. It is then converted to QUIN by the action of 3-hydroxyanthranilic acid dioxygenase (3-HAO) and finally degraded to nicotinamide adenine dinucleotide (NAD+) (23). In addition, 3-HK can produce Xanthurenic acid (XA) in the presence of KAT, and 3-HAA can produce picolinic acid (PIC) in the presence of 2-amino3-carboxymuconate-6-semialdehydedecarboxylase (ACMSD). XA is a regulator of glutamatergic synaptic transmission, which can directly or indirectly regulate metabolic Glu receptors (24). PIC is a non-selective metal ion chelator and neuroprotective agent, which can inhibit QUIN induced neurotoxicity, but the inhibitory effect is weaker than KYNA (25).

When QUIN is in a high expression state, microglia and neurons are restricted in their catabolism of NAD+, which in turn leads to cumulative neurotoxicity of QUIN, manifested in recurrent major depression patients having higher levels of KYN metabolites (26). QUIN is an NMDAR activator that enhances Glu release from neurons, leading to excess microenvironmental Glu concentrations and neurotoxicity, and can also induce selective apoptosis of novel glial cells. Meanwhile, prolonged exposure of neurons to elevated QUIN disrupts the structure of dendrites and reduces the immunoreactivity of microtubule-associated protein 2, thereby perpetuating neurodegenerative disease (27). In addition to this, QUIN-induced free radical generation and oxidative stress may also produce neurotoxicity. Its induction of lipid peroxidation is regulated by interaction with Fe^2+^, forming QUIN-Fe^2+^ complexes that promote ROS production (21).

#### 3.1.2 Neuroprotection branch

KYN generates KYNA in the mammalian CNS in the presence of KAT. among the four KATs, KAT II is mainly expressed in astrocytes and is responsible for most KYNA synthesis. KYNA exerts a neuroprotective effect against the excitotoxic and apoptotic effects of NMDAR due to its ability to antagonize this receptor (28). In addition, KYNA can antagonize α7-nicotinic acetylcholine receptor (α7-nAChR), reducing extracellular Glu and dopamine (DA) levels (29). And it plays a direct immunomodulatory role through the role of G-protein coupled receptor 35 (GPR35) and Aryl hydrocarbon receptor (30). However, high levels of KYNA can be detrimental, leading to low Glu levels that interfere with cognitive function and are associated with psychiatric disorders such as Schizophrenia, including memory deficits and reduced dopaminergic and glutamatergic neurotransmission (26).

#### 3.1.3 Relationship between quinolinic acid and kynurenic acid

There is a dynamic balance between activation and antagonism of NMDA receptors by QUIN and KYNA, and this balance ensures normal calcium and sodium influx into the glutamatergic postsynapse, thereby increasing synaptic plasticity and cell survival. QUIN and KYNA cannot pass through the BBB, which can indicate whether the integrity or function of the patient’s BBB is damaged. In addition, QUIN production is excessive under disease conditions, while KYNA cannot fully block QUIN. Moreover, LPS induced the expression of KMO rather than KATII, which proved that systemic inflammation stimulated the transfer of KP to the neurotoxicity branch (17). The agonistic and antagonistic metabolites produced by KP metabolism are balance. Once disturbed, it will cause functional and structural damage to the CNS. However, the over activation of KP will regulate the disorder of balance through negative feedback. Therefore, KP usually has both pathological mechanisms and compensatory mechanisms (31).

### 3.2 Serotonin pathway

About 1-2% of TRP is produced as 5-hydroxytryptophan (5-HTP) by tryptophan hydroxylase (TPH) and metabolized to serotonin (5-HT), an important monoamine neurotransmitter, by Aromatic Amino Acid Decarboxylase (AAAD), for which TRP is the only precursor substance. Serotonin reuptake transporter (SERT) is the only efficient transporter of 5-HT in a physiological state. Re-absorption of 5-HT in interstitial space can maintain the 5-HT system homeostasis by transferring 5-HT into intracellular inactivation. In addition, 5-HT is metabolized in two directions:(1 it is transformed into 5-hydroxyindoleacetic acid (5-HIAA) under the action of monoamine oxidase (MAO), which is a biomarker of low-level 5-HT in the brain; (2 it is metabolized into N-acetylserotonin (NAS) and melatonin.

There are two subtypes of TPH, including TPH1 and TPH2. TPH1 mainly exists in enterochromaffin cells (ECS), spleen, pineal gland, and thymus, and TPH2 completely exists in central neuronal cells (32). Up to 90% of the total 5-HT production comes from ECs in the gut and, to a lesser extent, from 5-HTergic neurons in the enteric nervous system (ENS) (33). 5-HT is related to cognition, emotion, feeding, and so on. it is critical for planning and decision-making, and plays an important role centrally and peripherally. Melatonin is a genetic susceptibility factor and an inhibitor of the increase of intestinal permeability induced by alcohol. The decrease of melatonin may mediate some diseases by changing the intestine. In addition, melatonin can also optimize mitochondrial function and drive the regulation of circadian rhythm, which is related to the changes in mental diseases (34).

### 3.3 Indole pathway

Most bacteria, fungi, and protozoa in the gastrointestinal tract are affected by the availability of TRP and can directly convert TRP into various molecules such as indole and their derivatives. Including tryptamine, indoleacetic-3-acid (IAA), indolepropionic-3-acid (IPA), indolelactic acid (ILA), indole-3-aldehyde (IAld), indoleacrylic acid (IA), indole-3-acetaldehyde (IAAld), etc (35). Most of these metabolites are ligands of AHR. Indole is formed by tryptophanase (TNAA), which is expressed in many Gram-negative and Gram-positive bacterial species, including *Escherichia coli, Clostridium* spp.*, and Bacteroides spp* (36). Under the action of indole, the expression of pro-inflammatory cytokines and anti-inflammatory cytokines can promote intestinal health and regulate the coordinated changes of intestinal homeostasis. Trp decarboxylation to produce tryptamines is extremely rare in bacteria. Only *Xenorhabdus nematophilus*, *Bacillus atrophaeus*, and *Lactobacillus bulgaricus* are considered to produce tryptamine (37). Tryptamine is a ligand of Trace amine associated receptors (TAARs) and sigma-2 receptors, and can induce ECs to release 5-HT. In addition, Interleukin-4-induced gene 1 (IL4I1) is a key enzyme of IP (38). It is highly expressed in mature dendritic cells (DCs) and can catalyze Trp to produce IAA and IALD (39). IL4I1 is a potent agonist of AHR because it can mediate the production of indole metabolites (31).

## 4 Factors regulating tryptophan metabolism

**Figure 2:**
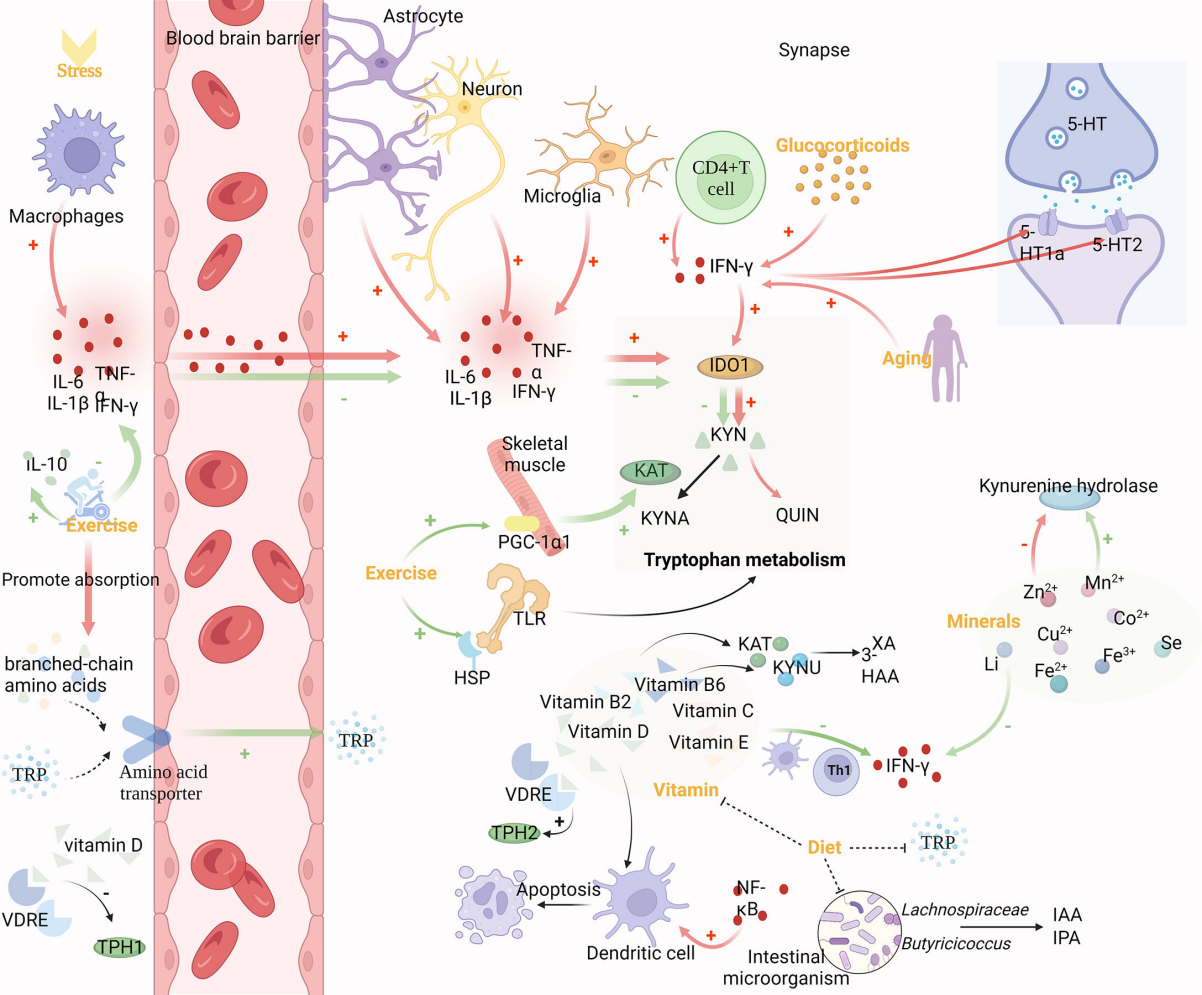
Mechanism of upstream regulation of TRP metabolism. Inflammation and stress, exercise, vitamins, diet and gut microbes, minerals, glucocorticoids and aging can modulate tryptophan metabolism. Pro-inflammatory cytokines produced by macrophages can cross the BBB and regulate IDO-1 expression together with pro-inflammatory cytokines produced by astrocytes, microglia, neurons, and CD4^+^ T cells in the brain. The binding of central 5-HT1a and 5-HT2 receptor sites is also influenced by cytokines like IFN-γ, which affects Glu release. Glucocorticoids and aging can enhance the activity of IFN-γ. Exercise decreases IDO-1 activity *via* anti-inflammatory effects and lessens branched-chain amino acids’ competitive effect on TRP entrance into the brain through the BBB, both of which result in an increase in TRP concentrations in the brain. Vitamins are directly involved in regulating in TRP metabolism as cofactors and coenzymes. Gut microbes promoting gut homeostasis, which also contributes to TRP metabolites and ω-3-fatty acids. Vitamins and gut microbes, which jointly govern tryptophan metabolism, can both be controlled by dietary changes. Additionally, minerals are cofactors and enzymes of KP, and KP enzymes are very sensitive to them.

Due to complex regulatory mechanisms, tryptophan metabolism is involved in various physiological mechanisms such as stress, inflammation, and KP metabolism. Therefore, understanding the multilevel control of this pathway can help predict susceptibility to related diseases and achieve multilevel effects from “mechanism-oriented therapy” to “disease-oriented therapy” (**Figure 2**).

### 4.1 Inflammation and stress

Pro-inflammatory cytokines are primarily produced by macrophages, but astrocytes, microglia, and neurons can also produce them in the brain. They can pass freely through the BBB by a variety of mechanisms, including passive diffusion through the leaky regions of the BBB, active transport, or *via* nerve fibers such as the vagus or trigeminal nerves (40). Pro-inflammatory cytokines are known to regulate KP enzymes. IDO-1 expression can be induced in several cell types by cytokines such as LPS, TNF, interleukin-1 (IL-1), and interleukin 2 (IL-2). Ibuprofen, a painkiller, boosted levels of the anti-inflammatory markers Arg-1 and YM-1, while decreasing the expression of TDO-2 in the process (41). In particular, IFN-γ released by activated CD4^+^T cells induced the expression of IDO-1, promoted the catabolism of TRP, and reduced the synthesis of central 5-HT (42). Not only that, but cytokines, such as IFN-γ also affect the binding of the central receptors 5-HT1a and 5-HT2 and increase the release of Glu, resulting in psychiatric disorders (43). Additionally, brain damage and high levels of oxidative stress might trigger the expression of pro-inflammatory cytokines. According to studies, chronic stress can significantly increase levels of interleukin 6 (IL-6) and TNF-α, which in turn results in large drops in TRP, 3-HAA, and indole, suggesting metabolic abnormalities in KP and IP (44).

### 4.2 Exercise

Aerobic exercise can reduce KYN levels in the circulation and central nervous system through muscle and other means. Long-term exercise has been demonstrated to prevent cognitive deterioration, reduce the level of pro-inflammatory cytokine IL-1β, and increase the level of anti-inflammatory cytokine IL-10 in brain tissue of mice with craniocerebral injury (45). Furthermore, IDO activity is decreased, the content of metabolic product KYN in the TRP/KYN pathway in the brain is reduced, and the concentration of free TRP is increased, so that it can enter the brain more through BBB (46). However, the transport of TRP through BBB depends on the ratio of TRP to branched-chain amino acids, which are more competitive than TRP in trans-BBB transport. Exercise alleviates this competition by increasing muscle absorption of branched-chain amino acids, thereby increasing TRP utilization in the brain (47). In addition, through exercise, skeletal muscle increases KAT expression and pushes the peripheral KP toward KYNA production (33). This effect may be related to the exercise-induced elevated expression of the PGC-1α1 in skeletal muscle cells. Pgc-1α1 effectively regulates KAT activity and protects the brain from the harmful effects of KYN accumulation (48). However, changes in TRYCATs could be detected after the toll-like receptor (TLR) family was activated (49). After acute exercise, the expression of heat shock proteins (HSPs) is upregulated, which may be due to the cross-tolerance of TLR4 and induce the decreased expression of TLR4 in tissue cells (50). Therefore, exercise can also affect TRP metabolism by regulating the TLR family.

### 4.3 Vitamins

Vitamins, which are necessary for cell growth and metabolism, operate as cofactors and coenzymes in the regulation of TRP metabolism and have an impact on the 5-HT receptor’s binding properties in adult rat brains (51). Studies have shown that vitamin D can affect TRP metabolism by affecting TPH1/2 (52). Two different vitamin D response elements (VDRE) exist in the regulatory regions of TPH1 and TPH2, which are responsible for converting TRP into 5-HT. Since VDRE responds differently to vitamins, vitamin D activates TPH2 in the brain and inhibits TPH1 outside of BBB (47). The study found that 58% of people who attempted suicide were vitamin D deficient, and their vitamin D levels were significantly lower than those of healthy individuals and depressed people who were not suicidal (53). Moreover, DCs can be activated by several pathways with NF-κB as the core. Vitamin D can inhibit the differentiation and maturation of immature DCs, inhibit the upregulation of CD40, CD80, and CD86, and promote the spontaneous apoptosis of mature DCs (54). In addition, vitamins B2 and B6 are decisive factors of the KP pathway, have a profound influence on the enzymes of KP, and are also markers of IFN-γ -mediated immune activation (55). Vitamin B2 is also a cofactor of KP, and the concentrations of XA and 3-HAA in plasma are positively correlated with vitamin B2 (56). Vitamin B6 is a cofactor of KYNU and KAT. In the KP process, except for 3-HK, all metabolites depend on vitamin B6 to produce, including 5-HT and melatonin (57–59). Vitamin B3 deficiency can lead to mental disorders, and this effect is mostly related to TRP metabolism. Mitogen can induce cell production of IFN-γ and further degradation of TRP. Vitamin C and vitamin E inhibit IFN-γ formation and release in a dose-dependent manner (60). This effect may be achieved by influencing the release of TH1-type cytokines by Tregs and DCs (61).

### 4.4 Diet and gut microbes

TRP comes from exogenous intake of protein-rich foods, including chicken, tuna, oats, peanuts, bananas, milk, cheese, and chocolate. Vitamin B6, on the other hand, is water-soluble and its intake can also be adjusted through food (58). Thus, the metabolic pathways are influenced by adjusting food intake to affect the source of metabolic pathway precursors and the activity of related enzymes. It was shown that a high protein diet enhances rat liver TDO activity in a dose-dependent manner, while a high carbohydrate diet increases brain TRP availability and a high-fat diet also inhibits rat liver TDO activity and promotes 5-HT synthesis by increasing brain TRP (55). In addition, changes in the microbiota regulate the host immune system by modulating TRP metabolism, and diet can influence the composition of the gut microbiota. Clinical trials have found that after a mediterranean diet (rich in plant-based foods such as fruits, vegetables, nuts, legumes, seeds, and grains, as well as olive oil, dairy, fish, and poultry), fiber-fermenting bacteria such as *Lachnospiraceae* and *Butyricicoccus* were added, and IAA and IPA (beneficial to nerve cells) generated by bacteria were significantly increased (62). With the intake of more dietary fiber, the anaerobic gut microbes produces more short-chain fatty acids (SCFA), promoting gut homeostasis, which also contributes to TRP metabolites and ω-3-fatty acids (63). And there is an association between higher levels of omega-3 fatty acids and lower levels of anxiety (64).

### 4.5 Minerals

Minerals such as Mn^2+^, Zn^2+^, Co^2+^, Cu^2+^, Fe^2+^, Li, and Se are cofactors and coenzymes of KP, to which the enzymes of this pathway are highly sensitive. It was found that Zn^2+^ inhibits kynurenine hydrolase, while Mn2+ activates this enzyme. Both metal ions activate KAT, while Cu^2+^ and Co^2+^ inhibit it, and these inhibitory effects are due to the blocking and inactivation of the sulfhydryl group of the enzyme (65). In addition, in the 5-HT pathway, TPH is strictly dependent on Fe^2+^ and is inactivated by Fe^3+^. Also, it has been found that the induction of the KP pathway by IFN-γ through increased IDO-1 expression can be inhibited by Li (66).

### 4.6 Glucocorticoids

Factors detrimental to mental health, such as stress, social isolation, sleep deprivation and lack of physical activity, raise circulating glucocorticoid levels in humans and non-human social mammals. Whereas glucocorticoids can enhance IDO-1 expression by enhancing INF-γ activity, not only that, glucocorticoids can also enhance TDO activity (67, 68). The researchers detected an increase, decrease, and increase in the ratios of KYN/TRP, KYN/QUIN, and KYN/KYNA in the serum of rats after administration of hydrocortisone. The data suggest that the administration of glucocorticoids resulted in the promotion of KP in rats and a shift of KP toward the production of QUIN (69).

### 4.7 Aging

Currently, studies on model organisms such as yeast, worms, flies and mice have found a relationship between TRP metabolism and aging and age-related diseases including Alzheimer’s disease, Parkinson’s disease and Huntington’s disease. Studies have found that the ratio of KYN/TRP increases in the elderly, and the degradation rate of TRP increases. IDO in the brain increases with age, and the elderly with higher KYN/TRP ratio have higher mortality (16). Aging alters KP metabolism and TRP availability, thereby increasing susceptibility to age-dependent neurological diseases (70). However, IDO activity in the liver and kidney decreased with age. The increase in inflammation observed during aging is the driving force of KP activity and leads to the overproduction of QUIN. In addition, the activity of IFN-γ increased with age, suggesting that increasing age may modulate IDO activity because IFN-γ can activate IDO. These data suggest that TRP metabolism is a powerful metabolic regulator of aging and age-related diseases, providing a new approach to disease intervention (71).

## 5 Regulation of TRP metabolism on downstream targets

**Figure 3:**
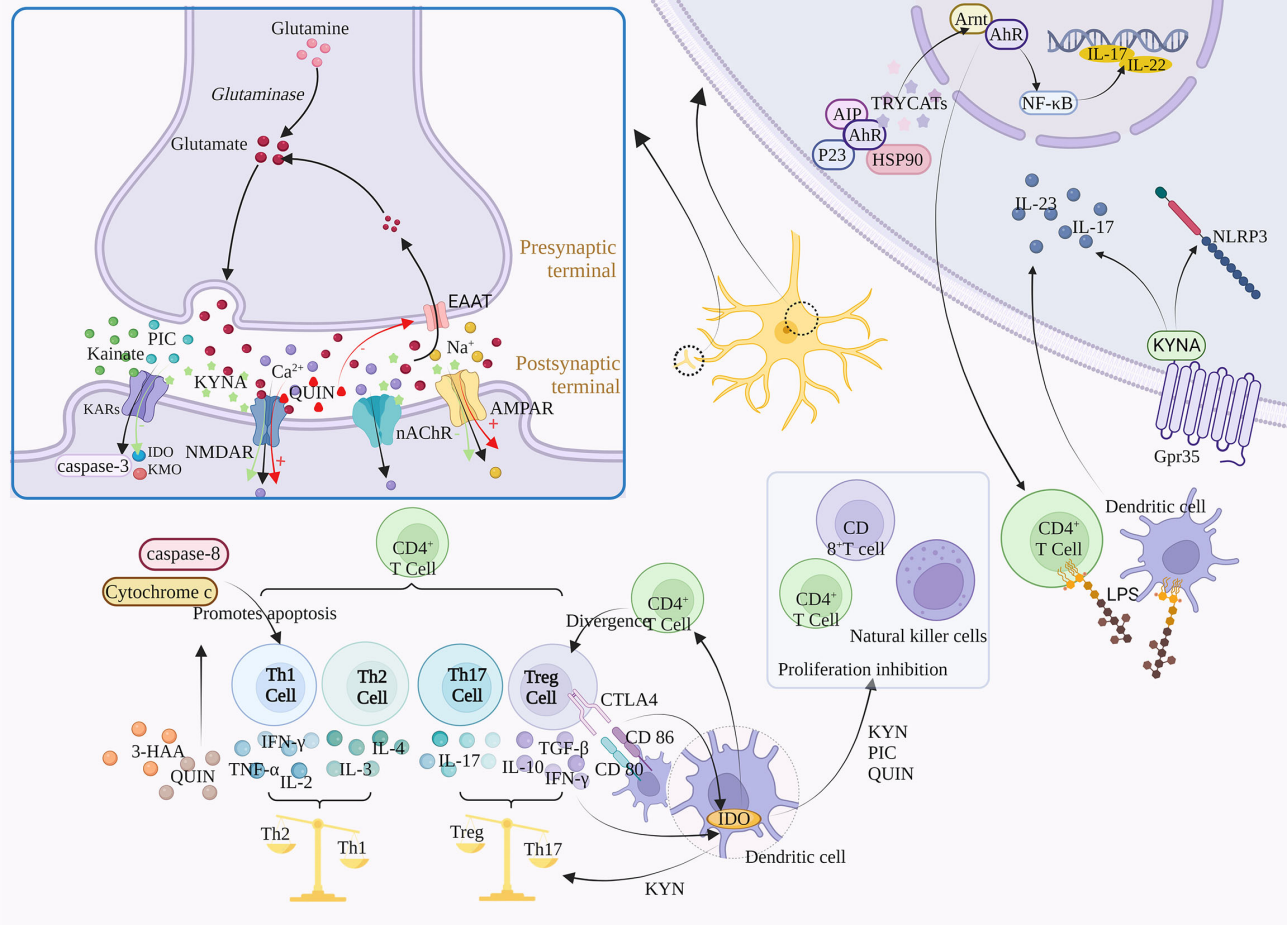
Regulatory mechanism of TRP metabolism on downstream signals. TRYCATs can achieve the regulation of Glu by acting on NMDAR, AMPAR, GABA, and KARs. For example, L-KYN, PIC and QUIN can exert immunosuppressive effects through different mechanisms, inhibiting the proliferation of CD4^+^ T cells, CD8^+^ T cells and natural killer (NK) cells. The specific activation of Gpr35 by KYNA can exert anti-inflammatory effects through negative regulation of NLRP3 and release of IL-23 and IL-17. α7-nACh receptors mediate large amounts of Ca^2+^ into neurons at resting or hyperpolarized membrane potentials, whereas NMDAR directs Ca^2+^ into neurons in a depolarized state. In addition, TRYCATs are the major endogenous ligands for AHR, which are released from the HSP90 complex and translocated to the nucleus when AHR binds to the ligand.

Tryptophan metabolism has numerous downstream targets that have been demonstrated. KYNA, for example, can affect the antagonism of NMDAR, α7-nAChR, AMPA, Kainate receptors, and activation of Gpr35. At high concentrations, KYNA antagonizes AMPA and Kainate receptors, and at low concentrations, inhibits α7-nAChR (72)(**Figure 3**).

### 5.1 Glutamate regulation s

Glutamate (Glu) is an excitatory neurotransmitter, and about 70-80% of neurons in the cerebral cortex are glutaminergic neurons. Glu is not only a direct precursor of the inhibitory neurotransmitter γ-aminobutyric acid, but also a potential neurotoxin in the brain. Excessive amounts can lead to neuronal death or brain damage, while low levels are associated with the development of Schizophrenia, suggesting that maintenance of Glu homeostasis is critical for neural activity (73). In addition, Glu regulates synaptic efficiency and controls the release of various biomolecules, including cytokines. Therefore, Glu may cause a variety of physiological/pathological conditions (74).

Glu is involved in the body’s physiological activities by acting on ionic receptors and metabolic receptors, among which ionic receptors include NMDA receptors, AMPA receptors, and kainate receptors, which mediate the excitatory transmission of Glu. The massive activation of Glu receptors leads to the imbalance of Ca^2+^ in cells, which activates a large number of proteases, such as nitric oxide synthase (NOS) and protein kinase A (PKA), and activates relevant signal pathways to mediate the excitatory toxicity of cells (75). QUIN and KYNA can affect Glu signal transduction in the brain. The abnormal production of these metabolites is related to neurodegeneration and other neurological diseases, including Depression, Bipolar disorder, Addiction and Schizophrenia.

#### 5.1.1 N-methyl-D-Aspartic acid

N-methyl-D-Aspartic acid receptor (NMDAR) is a subtype of ionic glutamate receptor. It is composed of NR1, NR2 (A, B, C, D), and NR3 (A, B), among which NR1 is the basic subunit constituting the ion channel (76). NMDAR activity follows a hormetic dose-response curve, meaning that too much or too little NMDA activity is harmful to neurons (77). Activation of this receptor is dependent on Glu binding to the NR2 subunit and glycine binding to the NR1 or NR3 subunit (78).

KYNA can competitively bind to Glu and glycine sites on the NMDAR, with a stronger affinity for the latter, such as the Gly-B site (Glycine site B) (79, 80). Moreover, the inhibition is reversible. KYNA is the only endogenous NMDAR antagonist, while most of the other KYN metabolites are NMDAR agonists (81). After NMDAR activation, QUIN leads to extracellular excess of Glu by inhibiting Glu reuptake, and further NMDAR excitation is induced (82). The activation of NMDAR leads to the blocking of the elongation phase of protein synthesis, thus inhibiting protein translation (83). In addition, excessive activation of NMDAR leads to pathologically open ligand-gated Ca^2+^ channels, resulting in massive extracellular Ca^2+^ influx and intracellular Ca^2+^ overload. The direct consequence of increased intracytoplasmic free Ca^2+^ concentration is the activation of various enzyme systems regulated by calcium under normal conditions, including proteases, oxidase systems, protein kinases, phospholipases, endonucleases, etc. It aggravates the damage and energy crisis of biofilm, and is closely related to the generation and damage of free radicals (84). In addition, activation of NMDAR leads to Na^+^, and K^+^ influx into cells, which then activates downstream signaling pathways and secondary messenger molecules, ultimately leading to various synaptic changes. Although QUIN and Glu have similar affinity for NMDAR, QUIN has a greater excitotoxicity potential due to its lower reuptake efficiency and longer retention time in the synaptic cleft (26).

#### 5.1.2 α-amino-3-hydroxy-5-methylisoxazole-4-propionic acid

α-amino-3-hydroxy-5-methylisoxazole-4-propionic acid receptor (AMPAR) is one of the main Glu receptors in the brain and is expressed in all glial cells. It is a tetramer composed of four subunits (GLIA1-GLIA4) that plays a key role in regulating excitatory synaptic transmission. The GliA2 subunit is a decisive factor in the calcium permeability of AMPA, and the AMPA receptor lacking GliA2 is mainly expressed in inhibitory intermediate neurons (85). Microglia and macrophages also express AMPA receptors, whose activation contributes to the release of pro-inflammatory cytokines. KYNA plays a dual role in the dose-dependent regulation of AMPA receptors, which are activated at low concentrations and neuroinhibitory at high concentrations (86). In addition, activation of AMPA receptors increased the firing rate of 5-HT neurons and the release of 5-HT in both local and terminal regions, and both 5-HT neurons showed concentration-dependent excitatory AMPA responses (87).

#### 5.1.3 γ-aminobutyric acid

GABAergic interneurons are inhibitory neurons, accounting for 20% of all cortical neurons, and inhibit the excitation of downstream glutamatergic neurons. The antagonistic effect of NMDAR prevents the activation of GABA neurons and leads to the disinhibition and Glu surge of downstream glutamatergic neurons, which activates the postsynaptic AMPAR and enhances brain-derived neurotrophic factor/tyrosine receptor kinase B (BDNF/TrkB) signaling pathway, ultimately increasing synaptic plasticity and synaptic strength (88). Submicromolar concentrations of KYNA antagonize nicotinic receptor function in the prefrontal cortex, thereby inhibiting GABAergic neurons. In contrast, the inhibitory effect of KYNA on GABA could be reversed by KAT II inhibitor, showing that KAT II activity could regulate GABA (89).

#### 5.1.4 Kainate receptors

Kainate (KA) is an agonist of Kainate receptors (KARs), and also acts as a non-desensitizing agonist of AMPA receptors. KARs, ligand-gated channel ionic receptors, exist in the postsynaptic membrane and mediate a small portion of ionic synaptic responses, playing a key role in synaptic integration (90). KARs is a tetramer composed of five subunits, including GluK1-5, of which GluK2 subunit is a key determinant of KARs properties. KA has excitatory toxicity, can activate caspase-3, and induce DNA fragments in hippocampal CA3 and CA1 regions, which is consistent with the occurrence of apoptotic cell death. However, inhibition of certain aspects of neuroinflammatory response ameliorates KA-induced neurotoxicity (91). Administration of KA induces the expression of IDO and KMO, but treatment with KMO inhibitors ameliorates KA-induced neurotoxicity (92). It was found that elevated KYNA levels antagonized the neurotoxicity of KA, while PIC modulated the KA-induced striatum Glu release (25).

### 5.2 Immune regulation

Th1, Th2, and Th17 cells belong to different Th cell subpopulations differentiated from CD4^+^T cells. Th1 cells can differentiate from CD4^+^T cells under the induction of cytokines such as IL-12 and mainly secrete IFN-γ, TNF-α, and IL-2 to exacerbate inflammation (93). In addition, Th2 can exert anti-inflammatory effects by secreting IL-4 and IL-3, and Th17 can secrete pro-inflammatory factors such as IL-17 to promote inflammation (94). Studies show that IDO is involved in immune tolerance and Th1/Th2 regulation, IDO-TRP metabolism, and Treg positively regulate one another, while Treg can release IL-10, and TGF-β to suppress inflammation (94). Tregs may induce IDO expression in DCs through the interaction between CTLA4 on Tregs and CD80/CD86 on DCs, or through cytokines secreted by Treg (e.g., IFN-γ). In contrast, IDO expression in DCs may induce differentiation of new Tregs from naive T cells. IDO was found to be a “switch” for the conversion of Th17 to Treg, inhibiting the differentiation of Th17 cells and increasing Treg, which may be related to KYN (95, 96). Kynurenines primarily induce a negative feedback loop and cell death in the Th1 cell population and promote upregulation of the Th2 cell population, which leads to a relative shift in the Th1-Th2 ratio toward Th2. In addition, 3-HAA and QUIN can induce selective apoptosis in Th1 cells through activation of caspase-8 and release of cytochrome c from mitochondria (97).

In addition, KP can affect T cell proliferation, and activation of IDO-1 in DCs completely prevents clonal expansion of T cells (98). Indeed, the negative feedback provided by IDO expression in DCs after contact with activated T cells is a necessary immunoregulatory mechanism of T cell deactivation (99). The kynurenine metabolites L-KYN, PIC, and QUIN can exert immunosuppressive effects through different mechanisms, inhibiting the proliferation of CD4^+^ T lymphocytes, CD8^+^ T lymphocytes, and NK cells. This T cell proliferation inhibition may be associated with a sustained cell cycle arrest, where IDO blocks cells in the mid-G1 phase, and this inhibitory effect is selective and only applies to cells that are being activated (100, 101). In contrast, activated CD4^+^ T cells can release TNF-γ, which induces LATs to transport TRP across the blood-brain barrier to the cytoplasm, maximizing TRP consumption in IDO-1-expressing cells, a process that involves the positive feedback mechanism of KYN-AhR (10). In addition, 3-HAA can lead to dysfunction and cell death of activated Th2 cells by inhibiting NF-κB. 3-HAA has been shown to directly inhibit the activity of Th1 and Th17 cells and also indirectly reduce their activity by increasing the amount of TGF-β secreted by DCs, leading to an increase in the number of Treg cells produced by primitive CD4^+^ cells.

### 5.3 G protein-coupled receptor

G protein-coupled receptors (GPCRs) are one of the largest families of genes that can be activated by a variety of ligands, most of which are metabolic intermediates, including 5-HT and melatonin produced by TRP metabolism. While orphanized G protein-coupled receptor 35 (Gpr35), an orphan receptor in GPCRs, can be specifically activated by its ligand KYNA and its expression is mainly associated with immune cells and the gastrointestinal tract (102). KYNA was found to have anti-inflammatory effects, and this effect was associated with its activation of GPR35. (1) promoting autophagy of NLRP3 inflammatory vesicles, thus exerting a negative regulatory effect on NLRP3 (103); and (2) inhibiting LPS-induced release of IL-23 and IL-17 from DCs and CD4^+^ T cells (104). In addition, KYNA regulates energy homeostasis in adipose tissue and cytokine release from invariant NK cells by activating Gpr35, which affects Ca^2+^ release, ERK1/2 phosphorylation, and Pgc-1α1 activation (105).

### 5.4 Nicotinic acetylcholine receptor

Nicotinic acetylcholine receptor (nAChR) belong to the cys-loop receptor superfamily, a ligand-gated ion channel protein with two subtypes, N1 (α1, β1, δ, ϵ, γ) and N2 (α2-α10, β2-β4) (106). Due to the different distribution regions, the N1-type receptors are called neural nicotinic receptors and the N2-type are called muscle nicotinic receptors. nAChR, similar to NMDAR, is involved in regulating neuroplasticity. α7-nACh receptors mediate large amounts of Ca^2+^ into neurons at resting or hyperpolarized membrane potentials, whereas NMDAR directs Ca^2+^ into neurons in a depolarized state. Furthermore, α7-nAChRs have synergistic effects with NMDAR, and the interaction between the cholinergic and glutamatergic systems is necessary to induce synaptic plasticity and BDNF dependence. α7-nAChRs are present at the postsynaptic sites of glutamatergic synapses, and acetylcholine acting on α7-nAChRs may provide permissive depolarization of the postsynaptic membrane, thereby increasing the number of the responsive NMDAR (107). Among brain nAChRs, α4β2-nAChR and α7-nAChR are the most prevalent, whereas α7-nAChR protects against Glu neurotoxicity and prevents cell death (108). KYNA regulates α4β2-nAChR expression by noncompetitive inhibition of α7-nAChR, and α7-nAChR was more sensitive to KYNA inhibition than NMDAR. In addition to the effect of KP metabolites on nAChR, melatonin produced by SP can also activate α7-nAChR, thus enabling the optimization of mitochondrial function (109).

### 5.5 Aryl hydrocarbon receptor

The aryl hydrocarbon receptor (AhR), often referred to as the dioxin receptor, is a ligand-activated transcription factor that is widely expressed centrally and systemically. AHR signaling is thought to be an important component of the barrier site immune response and can regulate a variety of cellular processes, including immune regulation, cell development, differentiation, proliferation, survival, and apoptosis (110, 111). AhR forms a protein complex with dimer of the 90 kDa heat shock protein (HSP90), AhR-interacting protein (AIP), the co-chaperone p23, and the protein kinase SRC, which is expressed in the cytoplasm (112). TRYCATs are the major endogenous ligands of AHR, and when AHR binds to the ligand, it is released from the HSP90 complex and translocates to the nucleus, where it binds to the AhR nuclear translocator (Arnt) to form a heterodimer (109). As a key regulator associated with immunity and inflammation, AhR has been implicated in a variety of biological processes including regulation of immune responses, maintenance of mucosal barrier function, intestinal homeostasis, and tumor development. Kynurenine metabolites such as KYN, KYNA, QUIN, and 3-HAA act as ligands to activate AhR and stimulate the expression of the downstream target such as interleukin-22 (IL-22) and interleukin-17 (IL-17) (113). Activated AhR induces the proliferation of regulatory T cells (Tregs) CD4^+^, CD25^+^ and suppresses the immune function of activated T cells (114). The limitations of single IDO/TDO-targeted drugs can be overcome by selective blockade of AhR. In addition, its activation is associated with activation of toll-like receptor 2 (TLR2) and signaling pathways such as NF-kB downstream and MAPKs, which can promote phosphorylation of p65/NF-kB, JNK/MAPK, p38/MAPK and ERK/MAPK pathways to regulate multiple inflammatory signaling pathways and further promote the production of pro-inflammatory mediators, including interleukin-1β (IL-1β) and interleukin-6 (IL-6) (10).

### 5.6 Dopamine

Dopamine (DA) is a neurotransmitter known to reduce KYNA in the brain.KYNA has two opposing effects on DA neurotransmissiom. (1 KYNA can achieve excitatory effects on DA neurons by blocking the glycine site of GABA afferents to NMDA receptors. Elevated levels of KYNA in the brain increase the activity of dopamine neurons in the ventral tegmental area (VTA) dopamine and increase the firing rate and burst firing activity of these neurons (115). (2 KYNA inhibits α7-nAChR-induced decrease in DA release on dopaminergic nerve endings (72).

## 6 TRP metabolism in neurological and psychiatric disorders

**Figure 4:**
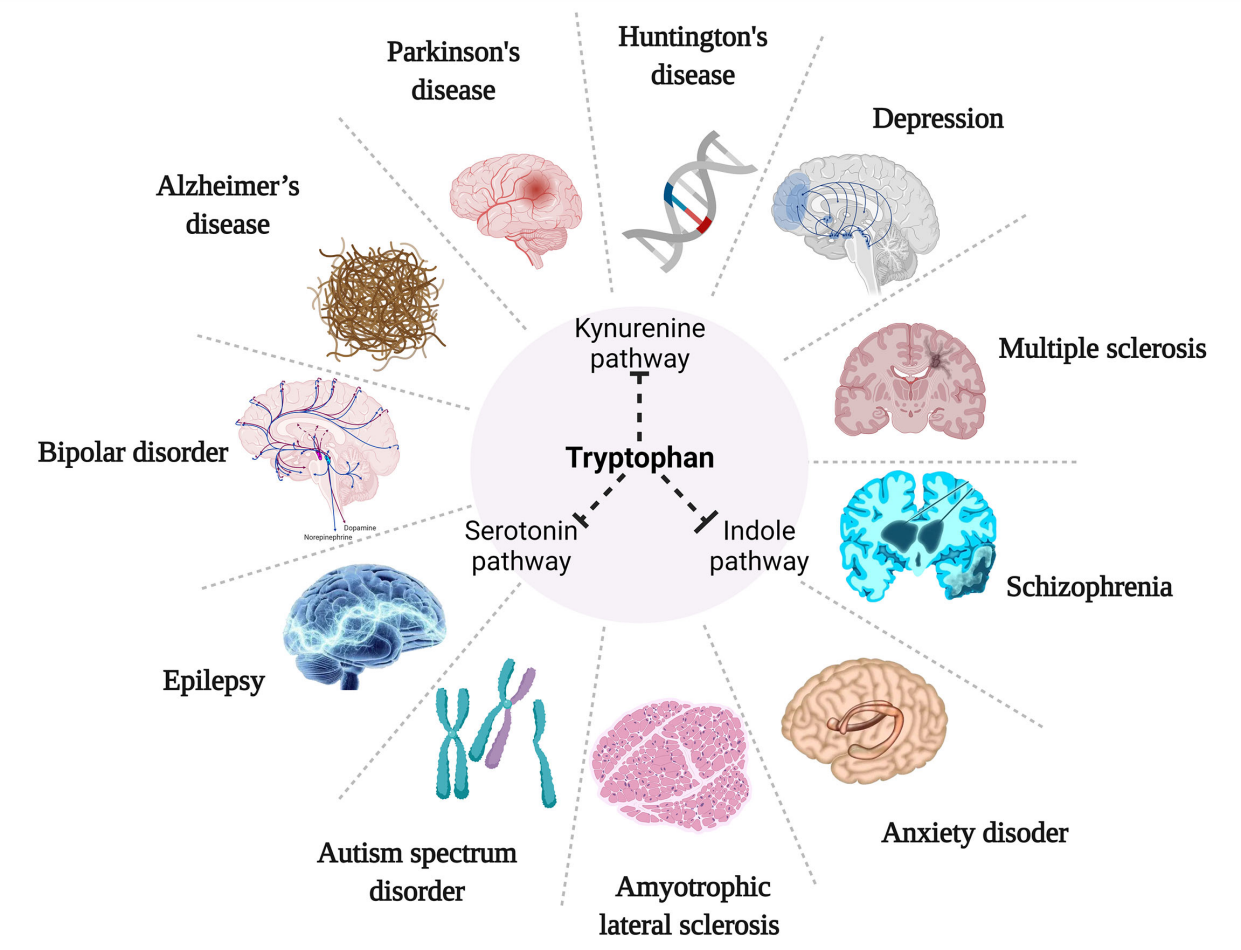
TRP metabolism for neurological and psychiatric disorders.

Alterations in TRP metabolism have been associated with neurological and psychiatric disorders, and TRYCATs alterations have also been found in MDD, cancer, diabetes, cardiovascular disease, autoimmune syndrome, and other diseases (**Table 1**). TRP metabolism demonstrates the greatest potential as the druggable target for neurological and psychiatric disorders. Therefore, this paper helps to guide the development of new drugs by summarizing current research on TRP metabolism in neurological disorders (Alzheimer’s disease, Parkinson’s disease, Huntington’s disease, Amyotrophic lateral sclerosis, Multiple sclerosis, Autism, Epilepsy) and psychiatric disorders (Depression, Schizophrenia, Anxiety, Bipolar disorder) (**Figure 4**).

**Table 1:** Neurological and psychiatric disorders and their risk factors are related to the changes in TRP catabolism and enzymes.

Disease	Sample	Alteration of metabolites	Sample	Alteration of enzymes	References
Alzheimer’s disease	Plasma	TRP, KYNA decreased and QUIN increased	The prefrontal cortex, hippocampal	IDO increased	(116, 117)
Parkinson’s disease	Plasma,cerebrospinal fluid	3-HK in plasma increased, 3-HAA and 5-HT/KYN decreased, KYNA and KYNA/KYN in cerebrospinal fluid decreased, and QUIN/KYNA increased	Plasma	KAT I and KAT II decreased	(118, 119)
Huntington’s disease	Serum	KYN increases, KYNA/KYN decreases	Putamen	KAT I and KAT II decrease, IDO activation	(120, 121)
Depression	Hippocampu	Lower ratios of KYNA/KYN, KYNA/3-HK, and KYNA/QUIN	Serum, prefrontal cortex, hippocampus, and cerebral cortex	TPH2, KATII/KMO mRNA decreased, KMO, IDO-1 upregulated	(122–124)
Multiple sclerosis	Prefrontal cortex、Hippocampus、Spinal cord、Spleen	TRP, KYN, and KYN/TRP in the prefrontal cortex, hippocampus, spinal cord, and spleen increased; 3-HK and QUIN in the Prefrontal cortex and hippocampus increased, while KYNA decreased	Plasma	KAT I and KAT II decreased	(125, 126)
Schizophrenia	Plasma	TRP decreases, KYN and KYN/TRP increase	Cerebral cortex	KMO, 3-HAO reduced	(127, 128)
Anxiety disorder	Plasma	KYN, KYN/TRP elevated	Center seam core	TPH2 decreased	(129, 130)
Amyotrophic lateral sclerosis	Cerebrospinal fluid, cortical	QUIN, IDO, TRP, KYN increased	Motor cortex, spinal cord	TPH decreased, IDO increased	(131, 132)
Autism spectrum disorder	Serum	TRP and 5-HT increased and KYNA decreased	–	KMO expression	(133, 134)
Epilepsy	Plasma	TRP, KYN higher, KYNA, 3-HK, KYNA/KYN lower	–	IDO Expression	(135, 136)
Bipolar disorder	Serum	TRP, KYNA, PIC, QUIN/TRP, and PIC/QUIN elevated	Serum	IDO-1 expression is upregulated	(137)

### 6.1 Neurological diseases

Neurological diseases are mostly accompanied by neuroinflammation and neuronal pathological damage in the brain with certain motor and non-motor impairments (138). For example, early amyotrophic lateral sclerosis (ALS) mediates motor neuron degeneration through glutamatergic excitotoxicity causing hyperexcitability of motor cortex neurons (139). Pathological studies have revealed that Parkinson’s patients are characterized by striatal dopaminergic neuron deficits and local neuroinflammation in the midbrain region, accompanied by chronic inflammation, oxidative stress, and other damage, and these events are usually associated with tryptophan metabolism (138). According to research, tryptophan metabolism is typically out of equilibrium when neurological illnesses like Alzheimer’s, Parkinson’s, Huntington’s, multiple sclerosis, amyotrophic lateral sclerosis, autism, and epilepsy advance (131, 135, 140–143).

Alzheimer’s disease (AD) neuropathology is characterized by progressive accumulation of beta-amyloid-beta (Aβ) oligomers and plaques in the brain, which leads to neurodegeneration in several regions of the brain (66). Aβ can induce the expression of inflammatory cytokines such as IL-1β, TNF-α, and enzymes in KP, including: IDO, KYNU, KAT I, KAT IV, etc. Following that, Aβ affects the expression of KYN metabolites such as 3-HK, 3-HAA, and QUIN, leading to neurological tissue damage, which later allows mechanisms such as Aβ re-accumulation, glial activation, and KP upregulation to further exacerbate neurodegenerative lesions (141). TRP/KYN and KYNA/TRP are significantly elevated in the frontal cortex, putamen, pars compacta of the substantia nigra, blood and cerebrospinal fluid of Parkinson’s patients (PD), especially in the periphery, and their elevation was significantly associated with advanced Parkinson’s (143, 144). In addition, studies have found that both KYN, as well as KYN/TRP in the urine of PD patients, increase as the disease progresses and can be used as a biomarker for PD (145). The collection of urine samples is also simpler than that of blood or cerebrospinal fluid, making urine detection a non-invasive and accessible method. Yilin Wang (111) found that TRP supplementation inhibited nuclear translocation of NF-κB in a PD model. However, With the use of AhR pathway inhibitors, TRP’s neuroprotective effects and its suppression of NF-κB were reversed. In addition, increased KYNA levels in the brain protect nigrostriatal dopamine neurons from QUIN-induced excitotoxic damage, and the decrease in KYNA in PD patients was accompanied by a decrease in KAT-I and KAT-II activity (146, 147). In patients with Huntington’s disease (HD), KYNA was found to be reduced, while 3-HK and QUIN were elevated. The susceptibility to NMDA-mediated neurotoxicity in HD was linked to KP activity by inhibition of IDO and TDO activity, which decreased 3-HK and QUIN production (142). In addition, ameliorates neurodegeneration in the drosophila model of HD by inhibiting KMO that increasing KYNA levels, while their effect on neurodegeneration was also demonstrated by direct feeding of KYNA and 3-HK (148).

In addition, TRP levels were reduced in serum and cerebrospinal fluid of patients with multiple sclerosis (MS). After the administration of INF-β, an increase in the L-KYN/TRP ratio was seen, indicating that the pathophysiology of MS may entail the impact of activated IDO-1 on TRP metabolism (140). Meanwhile, the impaired immunosuppressive activity of Treg cells against Th1 and Th17 cells has been shown to play a role in the pathogenesis of MS. The neurotoxicity mechanisms of QUIN are highly overlapping with those of amyotrophic lateral sclerosis (ALS) and motor neuro death. QUIN was significantly elevated in neurons and microglia in the cerebrospinal fluid, motor cortex, and spinal cord of ALS patients, while cerebrospinal fluid IDO, TRP, and KYN were significantly elevated, and the 3-HK/KYNA and QUIN/KYNA ratios tended to be elevated (131). Since QUIN and 3-HK may have neurotoxic and KYNA neuroprotective effects, these findings may reflect the impairment of neurotoxic compounds by KP (149). It has been found that both reduced and elevated NMDAR functions are associated with autism spectrum disorder (ASD), and its deviation in either direction leads to ASD, demonstrating the importance of a normal range of NMDAR function. NMDAR modulators can alleviate repetitive or hyperactive behaviors in addition to social behaviors (150). TRYCATs, including KYNA and QUIN, can exert regulatory effects on both AMPAR and NMDAR to achieve effects on ASD. The researchers found that IDO-1 levels, KYN/TRP ratio and pro-inflammatory cytokine levels were elevated in the serum and hippocampus of mice with epilepsy in both the acute and chronic phases following status epilepticus (SE), and this was reversed in IDO-1 knockout mice. This demonstrated that reducing the production of IDO-1dependent neurotoxic metabolites could suppress epilepsy, attenuate neuronal damage, and ultimately inhibit glial cell activation and pro-inflammatory cytokine production (135). Not only that, but kynurenine metabolites can affect epilepsy by modulating enhanced excitatory neurotransmission mediatesd to glutamate receptors, including NMDAR and AMPAR, a well-established hypothesis for the pathogenesis of epilepsy (151). In addition, vagal stimulation increases AA levels by modulating tryptophan metabolism, which serves to reduce the number and frequency of seizures, and the increased AA levels are associated with improved mood (152).

### 6.2 Psychiatric disorders

Psychiatric disorders are often accompanied by pathological features of abnormal neurometabolic, dysfunction of the dopaminergic system, and hypo glutaminergic function, and tryptophan metabolism is strongly associated with all three abnormal states. It has been found that the course of depression, schizophrenia, anxiety, and bipolar disorder is often accompanied by imbalances in tryptophan metabolism (26, 127, 129, 137).

Studies have shown that TRP metabolism may affect depression in two ways: (1) TRP depletion leading to 5-HT underproduction and (2) neurotoxicity of KYN metabolites. Currently, 5-HT insufficiency is one of the currently recognized mechanisms in the pathogenesis of depression (153). The researchers propose that the lack of 5-HT in depression is caused by a shunt of TRP metabolism from 5–HT formation to KYN formation. TPH2 expression is significantly reduced and IDO-1 expression is upregulated in serum, prefrontal cortex and hippocampus of depressed mice, showing a shift of TRP metabolism toward KYN, while SP is somewhat inhibited (81). In contrast, administration of 1-methyl-tryptophan (1-MT), a competitive inhibitor of IDO enzymes in mice, significantly reversed the depressive behavior (122). In addition, KYNA/KYN, KYNA/3-HK and KYNA/QUIN ratios were reduced in depressed patients, while KYNA/QUIN ratio was positively correlated with hippocampal volume and negatively correlated with the severity of depression. These data show an imbalance of neuroprotection and neurotoxicity in depressed patients (26). Clinical studies have found that plasma TRP levels are significantly lower in schizophrenic patients (Schizophrenia, SZ), while KYNA and KYN/TRP are significantly higher, KATII activity is increased, KYNA is elevated, and lower TRP levels are accompanied by lower white matter integrity (127). However, KYNA is involved in cognitive processes and excitatory neural network formation through its hype inhibitory effects on NMDAR and α7-nAChR, involving physiological processes such as learning, memory, and synaptic plasticity, triggering psychiatric symptoms and cognitive deficits (154, 155). There are limited clinical studies on TRP metabolism and anxiety disorders, Mary I. Butler (129) studied the plasma levels of TRP metabolites, including KYN, TRP, KYNA, KYN/TRP, and KYNA/KYN, in patients with social anxiety disorder (SAD). Increased activation of KP was found, and differences in metabolite concentrations may be associated with abnormal pro-inflammatory cytokines. Whereas the activation of KP in SAD seems to be preferentially directed towards KYNA synthesis, patients exposed to chronic stress due to repeated social interactions can shift downstream metabolism towards KYNA production not only by stimulating the conversion of TRP to KYN but also. In addition to this, tryptophan metabolism has a contribution to bipolar disorder (Bipolar disorder, BD). The importance of TRYCAT pathways in BD is at least partly as a result of their integration of peripheral inflammatory and IDO changes with changes in central neuronal regulation, driven by changes in glia responses and TRYCAT fluxes. Patients with BD had elevated IL-6, IL-1β, TNF-α, and IFN-γ. The concentrations of TRP, KYNA, and PIC in the cerebrospinal fluid and the ratios of QUIN/TRP and PIC/QUIN were significantly higher than those in healthy controls, and QUIN/TRP indicated elevated IDO-1 activity (137). And the cerebrospinal fluid PIC levels were lower in BD patients with a history of suicidal behavior than in patients without suicidal behavior (156).

## 7 Interventions for neurological and psychiatric disorders through TRP metabolism

There is a growing body of research on TRP metabolism, especially on the involvement of TRYCATs and enzymes in the physiopathological processes of neurological and psychiatric disorders. Although the specific mechanism of action is not yet fully understood, the regulation of key enzymes such as IDO and TDO to interfere with their enzymatic reactions may provide effective therapeutic strategies for the corresponding neurological and psychiatric disorders and provide theoretical guidance and experimental basis for the development of new drugs (157). Therefore, this paper summarizes the current drugs based on TRP metabolism for the treatment of neurological and psychiatric disorders, including natural drug extracts, prescriptions, biologics and other drugs (**Supplementary Table 1**).

## 8 Conclusions

Neurological and neurological disorders are important frontiers of neuroscience in this century, and the science of targeting TRP metabolic pathways has tremendous research potential. It can be modulated at multiple levels to exert neurological and psychoprotective effects, opening up possibilities and posing challenges for the development of medications for a variety of disorders.

TRP has many metabolic directions and complex regulatory mechanisms. Its metabolites also participate in many pathological processes, such as excitotoxicity, neuroinflammation, oxidative stress, mitochondrial damage, and so on, forming a complex regulatory network. However, the current understanding of this network is only the tip of the iceberg. In this paper, we try to summarize the network and its development in neurological and psychiatric diseases, and promote the subsequent more comprehensive study of the TRP metabolic network. Micro-regulation of the TRP pathway in the CNS is a key challenge for targeted therapies. This paper summarizes the factors regulating TRP metabolism (inflammation and stress, exercise, vitamins, minerals, diet and gut microbes, glucocorticoids, and aging) and the downstream (regulation of glutamate, immunity, Gpr35, nAChR, AhR, DA) role of TRP metabolism, and intends to provide a systematic introduction to its multi-linked regulation. However, there are multiple enzymes regulated in the tryptophan metabolic pathway, including IDO, TDO, KMO, KAT, KYUN, etc. Although there are a large number of preclinical studies on the treatment of neurological and psychiatric disorders by regulating TRP metabolism, most of them focus on the metabolite levels of 5-HT, TRP, and KYN, the expression of IDO, etc., and fewer studies on complex enzymes. As an important regulator of the TRP pathway, future efforts will require numerous researchers to conduct comprehensive studies on it using multidisciplinary techniques from molecular, cellular, and tissue to assess the role of enzymes in TRP metabolism.

## Author contributions

NL, RA and DL designed the study; SY, YL, AS, JD, YM, JiW, XQL, SYL, YLZ, JYW supplied materials and analytic tools; DL, SY and YL wrote the paper. All authors contributed to the article and approved the submitted version.

## Funding

This work was supported by the Key Laboratory of Modern Chinese Medicine Preparation of Ministry of Education of Jiangxi University of Traditional Chinese Medicine [grant numbers zdsys-202107].

## Conflict of interest

The authors declare that the research was conducted in the absence of any commercial or financial relationships that could be construed as a potential conflict of interest.

## Publisher’s note

All claims expressed in this article are solely those of the authors and do not necessarily represent those of their affiliated organizations, or those of the publisher, the editors and the reviewers. Any product that may be evaluated in this article, or claim that may be made by its manufacturer, is not guaranteed or endorsed by the publisher.
