# Supplementary material for: Tryptophan metabolism: Mechanism-oriented therapy for neurological and psychiatric disorders
Source: Front Immunol. 2022 Sep 8;13:985378. doi: 10.3389/fimmu.2022.985378 (PMC9496178; doi:10.3389/fimmu.2022.985378)
Supplement: Supplementary file 1 [file Table_1.docx]

**Table 1** Drugs and mechanisms of neurological and psychiatric disorders based on TRP metabolism

| Diseases | Drugs | Classification | Type of Research | Therapeutic effects | Mechanisms | References |
| --- | --- | --- | --- | --- | --- | --- |
| Alzheimer’s Disease | Coptisine | Extracts | Animal experiments | Prevented neuron loss, reduced amyloid plaque formation, and ameliorated impaired cognition | Inhibited IDO in the blood and decreased the activation of microglia and astrocytes | (158) |
|  | Oren-gedoku-to | Extracts | Cellular experiments | Inhibits the activity of IDO-1 | Inhibits Ido-1 activity by binding directly to heme iron or by occupying a putative TRP binding site | (159) |
| Parkinson's disease | 1-Methyltryptophan | Chemical drugs | Animal experiments | Restoration of endogenous antioxidant levels, SOD, catalase, GSH, and HO-1 | Inhibits IDO activity, reduces QUIN and 3-HK synthesis, and inhibits inflammation thereby reducing oxidative stress. Increased the activity of KAT-1, resulting in KYNA | (116) |
| Huntington’s disease | 2-(2-N, N-dimetilaminoetilamin-1-karbonil)-1Hkinolin-4-on-hidroklorid | Chemical drugs | Animal experiments | Increased body weight and the significant increase in striatal nerve cell volume prevented nerve cell atrophy | Neuroprotective effects similar to KYNA | (160) |
| Depression | Ginsenosides paradox | Extracts | Animal experiments | Improvements in anorexia and weight loss, depression-like behaviors such as sugar-water preference, hang-tail immobility time, and forced swimming immobility time. | Inhibition of central and peripheral IDO activity | (161) |
|  | Berberine | Extracts | Animal experiments | Increased mRNA expression of dopamine decarboxylase (DDC) pushes TRP toward 5-HT in the hippocampus and reduces the conversion of 5-HT to 5-HIAA | BB improved the stress-induced activation of IDO-1 and increased the KYN/TRP ratio. | (162) |
|  | Cang-Ai Volatile Oil | Extracts | Animal experiments | Improved depression-like behavior and decreased pro-inflammatory cytokine levels in the prefrontal cortex in rats. Decreased KYN and QUIN levels and the KYN/TRP ratio, while increasing TRP, KYNA, 5-HT, and 5-HIAA levels and decreasing the number of positive cells co-localized with microglia markers Iba-1 and IL-6 | Inhibits microglia activation and downregulates IDO expression, thereby inhibiting KP and reversing the effects on the 5-HT system | (163) |
|  | Sini Powder | Prescriptions | Animal experiments | It can shorten the immobility time and reduce serum IFN-γ levels in mice | Can inhibit IDO activity and mRNA expression quantity and regulate TRP-KYN metabolism | (164) |
|  | Tiansi liquid | Prescriptions | Animal experiments | Reduction of immobility time in mice | IDO activity and expression were regulated by inhibiting the direct and indirect induction of IDO by NF-κB. | (165) |
|  | Xiaoyaosan | Extracts | Animal experiments | Reversal of reduced sugar-water preference and improvement of depression-like behavior | Regulation of 5-HT and TRP concentrations mainly through upregulation of TPH2 expression and downregulation of IDO-1 activity | (166) |
|  | Curcumin | Extracts | Animal experiments | Improves depression-like behavior, reduces IL-1β, IL-6, and TNF-α mRNA expression and inhibits NF-κB activation in rats, inhibits stress-induced activation of the P2X7R/NLRP3 inflammatory axis, and reduces the conversion of pro-IL-1β to mature IL-1β | Inhibition of NLRP3 inflammasome and KP to alleviate depression-like states | (167) |
|  | Gentiopicroside | Extracts | Animal experiments | Improved depression-like behavior in mice and reduced the expression of IL-1β, TNF-α, and GluN2B subunits | Inhibited over-activation of IDO | (168) |
|  | Desipramine | Chemical drugs | Animal experiments | significantly reduced LPS-induced plasma IFN-γ protein | Blocking LPS-induced Ido1 expression in hippocampus, astrocytes, microglia, and human peripheral blood mononuclear cells (PBMC) and IDO-2 expression in PBMC | (169) |
|  | Glycyrrhizic acid | Extracts | Animal experiments | Improvement of depression-like behavior and inhibition of IDO activity in mice | Inhibits HMGB1 and thereby ameliorates chronic stress-induced depressive behavior by modulating KP | (170) |
|  | Hemerocallis citrina attenuate | Extracts | Animal experiments | Improvement of depression-like behavior and reversal of elevated IL-1β, IL-6, TNF-α levels, and IDO activity in rats | Restoration or improvement of the monoaminergic and neurotrophic systems exerting antidepressant effects | (171) |
|  | Peiyuan Jieyu Formula | Prescriptions | Animal experiments | Decreased immobility time and serum corticosterone levels and increased KYN/TRP ratio in mice | Anti-corticosterone and inhibit the production of neurotoxic metabolites of KP pathway to improve neuroprotective effects | (172) |
|  | Chaihu Shugan Decoction | Prescriptions | Animal experiments | Decreased immobility time in mice, inhibited IL-1β, TNF-α, and IDO mRNA expression in brain tissue, down-regulated KYN/TRP ratio, and increased 5-HT/TRP ratio | Inhibition of brain IL-1β and TNF-α levels, further inhibition of IDO mRNA expression, normalization of TRP metabolic pathway, and improvement of LPS-induced depression-like behavior in mice | (173) |
|  | Cajaninstilbene | Extracts | Animal experiments | Reversal of chronic unpredictable mild stress (CUMS)induced decrease in glycolytic preference index and increase in serum corticosterone level, and elevation of cortical DA, NE ,and Glu in mice | Lowering serum corticosterone levels and modulating neurotransmitters in the brain to achieve antidepressant effects | (174) |
|  | Cannabidiol | Extracts | Animal experiments | Improved depression-like behavior in mice, decreased the expression of TNF-α, IL-6, NF-κB, and reduced KYN/TRP, KYN/5-HT | Associated with a decrease in KP activation, IL-6 levels, and NF-ĸB activation | (5) |
|  | Asperosaponin VI | Extracts | Animal experiments | Improved depression-like behavior and reduced IBA-1 expression in mice | Inhibition of TLR4/NF-κB signaling pathway suppresses microglia-mediated neuroinflammatory responses and downregulates normalization of IDO expression and aberrant Glu transmission | (175) |
|  | Infliximab | Biological agents | Animal experiments | TNF-α, IDO, and HAAO expressions were downregulated and depression-like behavior was significantly reduced | Inhibition of TNF-α-mediated activation of IDO-HAAO pathway | (176) |
|  | Honokiol | Chemical drugs | Animal experiments | Reduced immobility time and pro-inflammatory cytokine levels in mice | Inhibits neuroinflammation by affecting NF-κB, decreases levels of pro-inflammatory cytokines, affect tryptophan metabolism, and increases neuroprotective metabolites | (177) |
|  | Fluoxetine | Chemical drugs | Animal experiments | Reduced resting time and depression-like behavior in animals forced to swim | Increased expression of TRP, 5-ht receptor, 5-HT1a, TPH | (178) |
|  | Baicalein | Extracts | Animal experiments、Cellular experiments | Shortened the duration of immobility in forced swimming, increased sucrose consumption, and restored stress-related dopamine concentrations in the hippocampus to near normal levels | Induction of stem cell neurite outgrowth and inhibition of IDO-1 protein activity | (179, 180) |
|  | *Chrysanthemum morifolium* | Natural Medicines | Animal experiments | Significantly increased sugar-water preference and serum 5-HT levels, and decreased corticosterone levels | Regulation of TRP metabolism, thus improving the secretion of 5-HT | (181) |
|  | Naringenin | Extracts |  |  | It regulates TRP metabolism, thereby improving 5-HT secretion, and exerts antidepressant effects by increasing the activity levels of TDO, IDO, and KAT. |  |
|  | Apigenin | Extracts |  |  |  |  |
|  | *Hypericum perforatum* | Extracts | Cellular experiments | Downregulate mitogenmediated TRP degradation | Inhibition of the activity of activated immunoreactive cells, resulting in reduced production of interferon-gamma | (182) |
|  | *Carthamus tinctorius* L. | Extracts | Cellular experiments | Inhibition of KP | Inhibition of IDO activity | (183) |
| Multiple sclerosis | *B.coagulans* | Bacteria | Animal experiments | Reduced expression of inflammatory factors (IL-4, TGF-β, IL-17, IFN-γ), IDO-1, CYP27B1, NLRP1, NLRP3, AIM2 genes, increased rate of remyelination and spatial memory in mice | Inhibition of Th1 and Th17 expression | (184) |
| Schizophrenia | Risperidone | Chemical drugs | Animal experiments | Regulation of 5-HT2a, 5-HT1b, 5-HT7, or alpha2C adrenoceptor | Reversal of Chronic TRP depletion-induced cognitive impairment | (185) |
| Anxiety Disorder | Ginsenosides paradox | Extracts | Animal experiments | Improves learning memory impairment and anxiety-like behavior, and increases TRP, 3-HK, 5-HT, and 5-HIAA levels | Regulation of TRP metabolism | (186) |
|  | Lavender oil | Extracts | Cellular experiments、Clinical Experiments | Anxiolytic effects and reduction of anxiety-related sleep disorders | Suppress mitogen-induced tryptophan degradation and IFN-γ production | (187, 188) |
|  | Itaconate | Biological agents | Animal experiments | Reducing anxiety-like behavior and attenuating neuroinflammation and impaired synaptic plasticity in the hippocampus of rats. | Restoration of hippocampal KP metabolic balance | (189) |
| Amyotrophic lateral sclerosis | Wedelolactone | Extracts | Animal experiments | Improvement of motor function and motor memory in rats | Increased Glutathione peroxidase activity and catalase activity, improving antioxidant activity. Effectively increased SOD activity and reduced nitrite production (wedelolactone reduced lipid peroxidation). Reduced LDH activity and l -glutamate level, inhibited caspase-3 activity | (190) |
|  | Gallic acid | Extracts | Animal experiments |  |  |  |
|  | N-acetyl-L-tryptophan | Chemical drugs | Animal experiments | Improved motor neuron loss and severe atrophy, and improved deterioration of motor performance | Reduced the release of cytochrome c/smac/AIF, increased Bcl-xL levels, inhibited the activation of caspase-3， ameliorated motor neuron loss and gross atrophy, and suppressed inflammation, as shown by decreased GFAP and Iba1 levels | (191) |
| Autism spectrum disorder | Tianeptine | Chemical drugs | Clinical Experiments | Transfer of atypical brain activation towards control levels during executive functioning (EF) in adult Autism spectrum disorder patients | Enhanced 5-HT reuptake, resulting in decreased plasma 5-HT and increased platelet 5-HT | (192) |
| Epilepsy | Quercetin | Extracts | Animal experiments | Reduced epilepsy severity score | Significant IDO inhibition and normalization of KYN/TRP levels in both the cerebral cortex and hippocampal fraction were observed. | (193) |
|  | Rhynchophylline | Extracts | Animal experiments | Reduces the severity of epilepsy and blocks spontaneous epileptiform discharges of neurons | Correction of NMDAR currents and altered NR2B protein expression | (131) |
|  | Asparagine | Extracts | Animal experiments | Improved seizure severity, the time span of seizures, and mortality | Inhibits NMDAR and prevents their neurotoxicity | (194) |
|  | Chaihu Shugan Decoction | Prescriptions | Animal experiments | Stopped depression-like behavior associated with chronic temporal lobe epilepsy and reduced the number of spontaneous seizures | Inhibition of brain IDO-1 activation | (195) |

158. Yu D, Tao B-B, Yang Y-Y, Du L-S, Yang S-S, He X-J, et al. The IDO inhibitor coptisine ameliorates cognitive impairment in a mouse model of Alzheimer's disease. *Journal of Alzheimer's Disease : JAD* (2015) 43(1):291-302.doi: 10.3233/JAD-140414

159. Yu C-J, Zheng M-F, Kuang C-X, Huang W-D, Yang Q. Oren-gedoku-to and its constituents with therapeutic potential in Alzheimer's disease inhibit indoleamine 2, 3-dioxygenase activity in vitro. *Journal of Alzheimer's Disease : JAD* (2010) 22(1):257-266.doi: 10.3233/JAD-2010-100684

160. Tóth F, Fülöp F, Szatmári I, Toldi J, Dékány I, Vécsei L. [Kynurenines and drug research]. *Orvosi Hetilap* (2020) 161(12):443-451.doi: 10.1556/650.2020.31673

161. Kang A, Hao H, Zheng X, Liang Y, Xie Y, Xie T, et al. Peripheral anti-inflammatory effects explain the ginsenosides paradox between poor brain distribution and anti-depression efficacy. *Journal of Neuroinflammation* (2011) 8(100.doi: 10.1186/1742-2094-8-100

162. Wang Q, Sun Y-N, Zou C-M, Zhang T-L, Li Z, Liu M, et al. Regulation of the kynurenine/serotonin pathway by berberine and the underlying effect in the hippocampus of the chronic unpredictable mild stress mice. *Behavioural Brain Research* (2022) 422(113764.doi: 10.1016/j.bbr.2022.113764

163. Zhang K, Lei N, Li M, Li J, Li C, Shen Y, et al. Cang-Ai Volatile Oil Ameliorates Depressive Behavior Induced by Chronic Stress Through IDO-Mediated Tryptophan Degradation Pathway. *Frontiers In Psychiatry* (2021) 12(791991.doi: 10.3389/fpsyt.2021.791991

164. Zhou J, Lu Y, Li H, Zhang J, Chang H. Antidepressant effects and IDO regulatory mechanism of Sini Powder on depression mouse induced by LPS. *China Journal of Traditional Chinese Medicine and Pharmacy* (2015) 30(12):4431-4433.doi:

165. Zhou J, Lu Y, Xu X, Zhang J, Li H, Chang H. Effect of antidepression and mechanism of regulation on IDO of Tiansi liquid *Journal of Beijing University of Traditional Chinese Medicine* (2015) 38(03):182-185.doi: 10.3969/j.issn.1006-2157.2015.03.008

166. Jiao H, Yan Z, Ma Q, Li X, Jiang Y, Liu Y, et al. Influence of Xiaoyaosan on depressive-like behaviors in chronic stress-depressed rats through regulating tryptophan metabolism in hippocampus. *Neuropsychiatric Disease and Treatment* (2019) 15(21-31.doi: 10.2147/NDT.S185295

167. Zhang W-Y, Guo Y-J, Han W-X, Yang M-Q, Wen L-P, Wang K-Y, et al. Curcumin relieves depressive-like behaviors via inhibition of the NLRP3 inflammasome and kynurenine pathway in rats suffering from chronic unpredictable mild stress. *International Immunopharmacology* (2019) 67(138-144.doi: 10.1016/j.intimp.2018.12.012

168. Deng Y-T, Zhao M-G, Xu T-J, Jin H, Li X-H. Gentiopicroside abrogates lipopolysaccharide-induced depressive-like behavior in mice through tryptophan-degrading pathway. *Metabolic Brain Disease* (2018) 33(5):1413-1420.doi: 10.1007/s11011-018-0246-y

169. Brooks AK, Janda TM, Lawson MA, Rytych JL, Smith RA, Ocampo-Solis C, et al. Desipramine decreases expression of human and murine indoleamine-2,3-dioxygenases. *Brain, Behavior, and Immunity* (2017) 62(219-229.doi: 10.1016/j.bbi.2017.02.010

170. Wang B, Lian Y-J, Dong X, Peng W, Liu L-L, Su W-J, et al. Glycyrrhizic acid ameliorates the kynurenine pathway in association with its antidepressant effect. *Behavioural Brain Research* (2018) 353(250-257.doi: 10.1016/j.bbr.2018.01.024

171. Liu X-L, Luo L, Liu B-B, Li J, Geng D, Liu Q, et al. Ethanol extracts from Hemerocallis citrina attenuate the upregulation of proinflammatory cytokines and indoleamine 2,3-dioxygenase in rats. *Journal of Ethnopharmacology* (2014) 153(2):484-490.doi: 10.1016/j.jep.2014.03.001

172. Chang H, Wang P, Liu B. Antidepressant effect of Peiyuan Jieyu Formula on acute and chronic stress BALB/c mice and regulation of TRP-KYN metabolic pathway. *Chinese Journal of Pharmacology and Toxicology* (2016) 30(10):1018.doi:

173. Cai L, Hu Y, Tan L, Liang J, Wei L. Effects of Chaihu Shugan Decoction on tryptophan metabolic pathway in acute depressive mouse induced by lipopolysaccharide. *China Journal of Traditional Chinese Medicine and Pharmacy* (2018) 33(11):5212-5215.doi:

174. Zhang M, Wang L, Li C, Tao X, Zhou Y, Liu X, et al. Antidepressant effects of cajaninstilbene acid on chronic unpredictable mild stress-induced depressive mice. *Acta Laboratorium Animalis Scientia Sinica* (2019) 27(01):85-90.doi: 10.3969/j.issn.1005-4847.2019.01.014

175. Zhang J, Yi S, Li Y, Xiao C, Liu C, Jiang W, et al. The antidepressant effects of asperosaponin VI are mediated by the suppression of microglial activation and reduction of TLR4/NF-κB-induced IDO expression. *Psychopharmacology* (2020) 237(8):2531-2545.doi: 10.1007/s00213-020-05553-5

176. Fu X-Y, Li H-Y, Jiang Q-S, Cui T, Jiang X-H, Zhou Q-X, et al. Infliximab ameliorating depression-like behavior through inhibiting the activation of the IDO-HAAO pathway mediated by tumor necrosis factor-α in a rat model. *Neuroreport* (2016) 27(13):953-959.doi: 10.1097/WNR.0000000000000637

177. Zhang B, Wang P-P, Hu K-L, Li L-N, Yu X, Lu Y, et al. Antidepressant-Like Effect and Mechanism of Action of Honokiol on the Mouse Lipopolysaccharide (LPS) Depression Model. *Molecules (Basel, Switzerland)* (2019) 24(11).doi: 10.3390/molecules24112035

178. Nadeem, Usman S, Imad R, Nisar U, Khan I, Abbas G. Pre-weaning fluoxetine exposure caused anti-depressant like behavior at adulthood via perturbing tryptophan metabolism in rats. *Metabolic Brain Disease* (2022).doi: 10.1007/s11011-022-00951-4

179. Chen S, Corteling R, Stevanato L, Sinden J. Natural inhibitors of indoleamine 3,5-dioxygenase induced by interferon-gamma in human neural stem cells. *Biochemical and Biophysical Research Communications* (2012) 429(1-2):117-123.doi: 10.1016/j.bbrc.2012.10.009

180. Lee B, Sur B, Park J, Kim S-H, Kwon S, Yeom M, et al. Chronic administration of baicalein decreases depression-like behavior induced by repeated restraint stress in rats. *The Korean Journal of Physiology & Pharmacology : Official Journal of the Korean Physiological Society and the Korean Society of Pharmacology* (2013) 17(5):393-403.doi: 10.4196/kjpp.2013.17.5.393

181. Liu T, Zhou N, Xu R, Cao Y, Zhang Y, Liu Z, et al. A metabolomic study on the anti-depressive effects of two active components from. *Artificial Cells, Nanomedicine, and Biotechnology* (2020) 48(1):718-727.doi: 10.1080/21691401.2020.1774597

182. Winkler C, Wirleitner B, Schroecksnadel K, Schennach H, Fuchs D. St. John's wort (Hypericum perforatum) counteracts cytokine-induced tryptophan catabolism in vitro. *Biological Chemistry* (2004) 385(12):1197-1202.doi:

183. Kuehnl S, Schroecksnadel S, Temml V, Gostner JM, Schennach H, Schuster D, et al. Lignans from Carthamus tinctorius suppress tryptophan breakdown via indoleamine 2,3-dioxygenase. *Phytomedicine : International Journal of Phytotherapy and Phytopharmacology* (2013) 20(13):1190-1195.doi: 10.1016/j.phymed.2013.06.006

184. Sadeghirashed S, Kazemi F, Taheri S, Ebrahimi MT, Arasteh J. A novel probiotic strain exerts therapeutic effects on mouse model of multiple sclerosis by altering the expression of inflammasome and IDO genes and modulation of T helper cytokine profile. *Metabolic Brain Disease* (2022) 37(1):197-207.doi: 10.1007/s11011-021-00857-7

185. Jenkins TA, Elliott JJ, Ardis TC, Cahir M, Reynolds GP, Bell R, et al. Tryptophan depletion impairs object-recognition memory in the rat: reversal by risperidone. *Behavioural Brain Research* (2010) 208(2):479-483.doi: 10.1016/j.bbr.2009.12.030

186. Bao Y, Chen Y, Zeng G, Yang Z, Pan R, Shi Z, et al. Protective Effect of Total Ginsenoside Ginseng Root on Learning and Memory Impairment and Anxiety in Rats Induced by Hindlimb Suspension. *Chinese Journal of Experimental Traditional Medical Formulae* (2021) 27(07):49-56.doi: 10.13422/j.cnki.syfjx.20210405

187. Gostner JM, Ganzera M, Becker K, Geisler S, Schroecksnadel S, Überall F, et al. Lavender oil suppresses indoleamine 2,3-dioxygenase activity in human PBMC. *BMC Complementary and Alternative Medicine* (2014) 14(503.doi: 10.1186/1472-6882-14-503

188. Kasper S, Gastpar M, Müller WE, Volz H-P, Möller H-J, Dienel A, et al. Silexan, an orally administered Lavandula oil preparation, is effective in the treatment of 'subsyndromal' anxiety disorder: a randomized, double-blind, placebo controlled trial. *International Clinical Psychopharmacology* (2010) 25(5):277-287.doi: 10.1097/YIC.0b013e32833b3242

189. Tang Y. ANTAGONISTIC ROLES OF ITACONATE IN B2-MICROGLOBULIN-INDUCED DEPRESSION- AND ANXIETY-LIKE BEHAVIORS AND THE UNDERLYING MECHANISMS: Nanhua University, 2020.

190. S M, T P, Goli D. Effect of wedelolactone and gallic acid on quinolinic acid-induced neurotoxicity and impaired motor function: significance to sporadic amyotrophic lateral sclerosis. *Neurotoxicology* (2018) 68(doi: 10.1016/j.neuro.2018.06.015

191. Li W, Fotinos A, Wu Q, Chen Y, Zhu Y, Baranov S, et al. N-acetyl-L-tryptophan delays disease onset and extends survival in an amyotrophic lateral sclerosis transgenic mouse model. *Neurobiology of Disease* (2015) 80(doi: 10.1016/j.nbd.2015.05.002

192. Wichers RH, Findon JL, Jelsma A, Giampietro V, Stoencheva V, Robertson DM, et al. Modulation of atypical brain activation during executive functioning in autism: a pharmacological MRI study of tianeptine. *Molecular Autism* (2021) 12(1):14.doi: 10.1186/s13229-021-00422-0

193. Singh T, Kaur T, Goel RK. Adjuvant quercetin therapy for combined treatment of epilepsy and comorbid depression. *Neurochemistry International* (2017) 104(27-33.doi: 10.1016/j.neuint.2016.12.023

194. Wong S-B, Hung W-C, Min M-Y. The Role of Gastrodin on Hippocampal Neurons after N-Methyl-D-Aspartate Excitotoxicity and Experimental Temporal Lobe Seizures. *The Chinese Journal of Physiology* (2016) 59(3):156-164.doi: 10.4077/CJP.2016.BAE385

195. Ren Z, Xie Y, Liu Y, Zheng Y. Chaihushugan Decoction Improves Epilepsy- associated Depressive- like Behavior in Rats with Chronic Temporal Lobe Epilepsy by Inhibiting of Brain Indoleamine 2,3-dioxygenase. *Lishizhen Medicine and Materia Medica Research* (2015) 26(05):1031-1034.doi: 10.3969/j.issn.1008-0805.2015.05.003
